# Decreased life expectancy: a health outcome not corrected by kidney replacement therapy that emphasizes the need for primary prevention of CKD

**DOI:** 10.1093/ckj/sfae053

**Published:** 2024-03-20

**Authors:** Lucia Cordero, Alberto Ortiz

**Affiliations:** Department of Nephrology and Hypertension, IIS-Fundacion Jimenez Diaz UAM, Madrid, Spain; Department of Nephrology and Hypertension, IIS-Fundacion Jimenez Diaz UAM, Madrid, Spain; RICORS2040; Madrid, Spain; Departamento de Medicina, Facultad de Medicina, Universidad Autónoma de Madrid, Madrid, Spain

The European Renal Association (ERA) Registry Annual Report 2021 was published online in 2023 [1]. Its data provide a compelling illustration of the need to prevent chronic kidney disease (CKD) to effectively improve outcomes, given the shortened life expectancy in patients undergoing kidney replacement therapy (KRT) as compared with the general population.

The ERA Registry Annual Report 2021 [1] illustrates the shortcomings of the current approach to kidney health. In most patients, the current criteria to diagnose CKD identify late stages of a disease process that, by the time CKD is diagnosed, has already wiped out 50% of the functioning kidney mass or caused injury leading to an increase in urinary albumin excretion of >10-fold the physiological level [2]. Increased albuminuria may allow earlier detection of CKD than low glomerular filtration rate (GFR), as it allows a CKD diagnosis and treatment when GFR is still normal. However, albuminuria assessment is underused and guidelines recommending albuminuria assessment in persons with diabetes mellitus, hypertension, the elderly and other high-risk populations are not followed. In fact, albuminuria assessment is not reimbursed outside of diabetes in some European countries such as Belgium, effectively impeding early diagnosis and treatment of CKD. This is clearly not helpful in addressing the high KRT burden. In this regard, the high incidence of KRT in Belgium [180–207 per million population (pmp)] lags behind Cyprus, Greece, Portugal, Tunisia and the Czech Republic, i.e. countries with a gross domestic product per capita that is 35–92% lower.

The fact that current diagnostic criteria frequently result in a delayed diagnosis of CKD as compared with the timing of initiation of kidney injury may be compensated by interventions aimed at actively preventing CKD. However, the concept of prevention, especially that of primary prevention, is underdeveloped for CKD. Primary prevention involves identifying persons at high risk of developing a condition (e.g. CKD) and actively intervening to decrease this risk. Post hoc analyses of three independent cardiovascular outcome clinical trials for sodium–glucose co-transporter 2 inhibitors in type 2 diabetes mellitus (T2DM) totalling 20 156 participants without baseline CKD, as assessed by estimated glomerular filtration rate (eGFR) and albuminuria criteria, have consistently demonstrated that CKD can be prevented in T2DM [3–5]. However, current guidelines do not discuss primary prevention of CKD in this context [6]. Thus the 2022 diabetes management in CKD consensus report by the American Diabetes Association and Kidney Disease: Improving Global Outcomes (KDIGO) indicates that for patients with a GFR >60 ml/min/0.73 m^2^ and A1 albuminuria the recommendation is ‘screen’, i.e. it fails to recommend a specific therapeutic intervention aimed at preventing new-onset CKD. In contrast, for patients who have already developed CKD, the recommendation is ‘treat’.

In 2021, the incidence of KRT in participating European countries was 145 pmp, 12% higher than in 2020 [7], with a majority (55% and 64%, respectively) of patients being ≥65 years of age and male [1]. The prevalence of KRT was 1040 pmp, 12% higher than in 2020 [7]. The increasing year-on-year incidence and prevalence of KRT further support the suboptimal results of current therapeutic approaches to CKD and illustrate the need for a holistic primary prevention road map. The most common primary renal disease was, once more, diabetes (22%). These data already pinpoint a high-risk population for whom prevention efforts should start 10–15 years earlier than age 65 years. Given the insidious nature of the onset of T2DM, universal assessment of cardiorenal risk using the ABCDE (albuminuria, blood pressure, cholesterol diabetes, eGFR) approach at age 50 years seems reasonable to identify individuals at risk for CKD (and cardiovascular disease), allowing timely preventive intervention [8, 9].

Diagnostic criteria for CKD are based on an increased risk of several adverse outcomes, including all-cause death [2]. Since current therapies for CKD do not bring this risk back to baseline levels, it is not surprising that mortality from CKD continues to increase worldwide [10]. Despite the widespread belief among the general population, healthcare planners and even some healthcare workers that KRT actually replaces all kidney functions to the point that the health issues related to CKD are all but solved, this is not the case. Life expectancy of persons on KRT is shorter than for the general population. In fact, for persons 20–24 years of age on dialysis, remaining life expectancy is 40 years shorter than in the general population of the same age [7, 10]. The increased risk of premature death is also not fully corrected by kidney transplantation: remaining life expectancy of a 20- to 24-year-old kidney transplant recipient is still 15 years shorter than in the general population of the same age. The ERA Registry Annual Report 2021 has gone one step further than prior iterations, providing gender-separated remaining life expectancy data for persons on dialysis or transplantation as compared with people of similar ages in the general population [1]. Th expected remaining lifetime for all age groups combined was 65–68% less for patients on dialysis and 40–43% less for kidney graft recipients than in the general population (Fig. 1). The largest loss of life expectancy was observed in young adults, peaking at 36 years shorter in 20- to 24-year-old men on dialysis and 16 years shorter if living with a functioning graft than in men from the general population. The corresponding female figures were even more concerning: 42 and 19 years shorter, respectively. The numbers remain concerning even at older age: life expectancy was 9 years (dialysis) and 6 years (transplant) (45%) shorter in elderly men and even shorter in elderly women (11 and 8 years shorter, respectively) than in elderly people in the general population. A general population woman who is 74 years old in 2024 in Europe would be expected to live until 2034, but only to 2026 if she is on dialysis. It should be noted that there are considerable intercountry differences, which may be even larger for countries not providing data for this analysis. Country-by-country data on life expectancy on KRT as compared with the general population should be generated in future iterations of registry data summaries, as this will provide a further benchmarking tool for the combined quality of healthcare systems and KRT across Europe.

**Figure 1:**
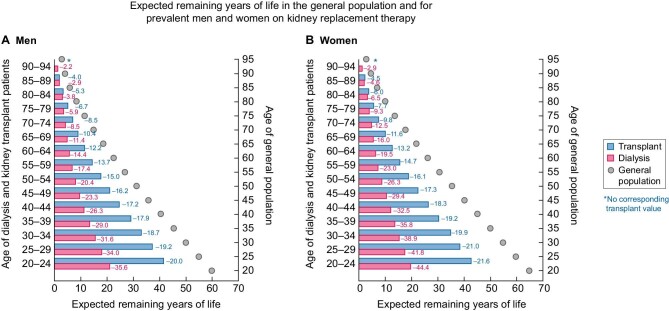
Despite KRT, the risk of premature death associated with CKD remains increased in patients with kidney failure. Expected remaining years of life in the general population and for prevalent men and women on dialysis or kidney transplant patients (cohort 2017–2021), reproduced from Figure 13 in Boerstra *et al.* [1]. The difference in years between life expectancy in the general population and patients on dialysis or with a functioning kidney graft for each age range is shown as a number. **(A)** Expected remaining years of life in men on KRT and in the general population. **(B)** Expected remaining years of life in women on KRT and in the general population.

In conclusion, the ERA Registry Annual Report 2021, as in prior reports, continues to provide a wealth of food-for-thought data that may contribute to reimagining care to preserve kidney health in the future. In this regard, the current strategy of initiation of CKD therapy once it has developed is associated with poor outcomes, including the increasing need for KRT and poor survival outcomes of persons on KRT when compared with the general population, emphasizing the need to increase the population-wide assessment of albuminuria as a means of diagnosing early CKD and of developing effective primary prevention strategies modelled after the cardiovascular disease success story.

## FUNDING

Funding was received from FIS/Fondos FEDER (PI22/00469, PI22/00050, PI21/00251), ERA-PerMed-JTC2022 (SPAREKID AC22/00027), Sociedad Española de Nefrología, Sociedad Madrileña de Nefrología (SOMANE), FRIAT, Comunidad de Madrid en Biomedicina P2022/BMD-7223, CIFRA_COR-CM, Instituto de Salud Carlos III (ISCIII) RICORS program to RICORS2040 (RD21/0005/0001) funded by European Union—NextGenerationEU, Mecanismo para la Recuperación y la Resiliencia (MRR) and SPACKDc PMP21/00109, FEDER funds; COST Action PERMEDIK CA21165, supported by COST (European Cooperation in Science and Technology); PREVENTCKD Consortium (Project ID: 101101220); Programme: EU4H DG/Agency: HADEA; KitNewCare (Project ID: 101137054); Call: HORIZON-HLTH-2023-CARE-04, Programme: HORIZON, DG/Agency: HADEA.

## CONFLICT OF INTEREST STATEMENT

A.O. is a former Editor-in-Chief of *CKJ* and has received grants from Sanofi and consultancy or speaker fees or travel support from Adviccene, Alexion, Astellas, AstraZeneca, Amicus, Amgen, Boehringer Ingelheim, Fresenius Medical Care, GSK, Bayer, Sanofi-Genzyme, Menarini, Mundipharma, Kyowa Kirin, Eli Lilly, Freeline, Idorsia, Chiesi, Otsuka, Novo Nordisk, Sysmex and Vifor Fresenius Medical Care Renal Pharma and Spafarma and is Director of Catedra UAM–AstraZeneca of chronic kidney disease and electrolytes. He holds stock in Telara Farma. L.C. declares no conflicts of interest.
